# Constipation in Older Adults: Pathophysiology, Clinical Impact, and Management Strategies

**DOI:** 10.3390/geriatrics11020047

**Published:** 2026-04-16

**Authors:** Shima Mimura, Asahiro Morishita, Atsuo Kitaoka, Kota Sasaki, Hiroki Tai, Rie Yano, Mai Nakahara, Kyoko Oura, Tomoko Tadokoro, Koji Fujita, Joji Tani, Takashi Himoto, Hideki Kobara

**Affiliations:** 1 Departments of Gastroenterology and Neurology, Faculty of Medicine, Kagawa University, Takamatsu 761-0793, Japan; 2 Division of Clinical Nutrition & Food Services, Kagawa University, Takamatsu 761-0793, Japan; 3 Department of Medical Technology, Kagawa Prefectural University of Health Sciences, Takamatsu 761-0123, Japan

**Keywords:** constipation, older adults, geriatric syndrome, central and autonomic dysregulation, brain–gut axis, frailty, healthcare burden

## Abstract

**Background/Objectives:** Constipation is a common gastrointestinal problem in older adults and is associated with reduced quality of life, functional decline, frailty, and an increased risk of delirium and cognitive impairment. Its pathogenesis is multifactorial, involving age-related changes in gastrointestinal motility, neural regulation, comorbidities, and polypharmacy. However, this condition has traditionally been regarded as a localized gastrointestinal disorder, which may not fully reflect its systemic clinical significance in older populations. While prior narrative reviews have described multifactorial contributors to constipation, none have formally applied a geriatric syndrome framework to integrate these dimensions. This review proposes a three-criterion operational definition—multifactorial pathogenesis, association with functional decline and frailty, and contribution to adverse systemic outcomes—to characterize constipation in older adults as a “systemic geriatric syndrome,” and evaluates available evidence against each criterion. **Methods:** A narrative literature search was conducted using PubMed to identify relevant studies published between 1 January 2023, and 31 December 2025. MeSH terms included “Constipation” [Major Topic] and “Aged” [MeSH Terms]. Eligible articles included English-language original studies, systematic reviews, and clinical or epidemiological studies involving individuals aged ≥65 years. **Results:** Diagnosis in older adults is often complicated by secondary causes, including medications and neurological disorders, as well as atypical symptom presentations in individuals with cognitive impairment. Key pathophysiological mechanisms include reductions in interstitial cells of Cajal, impaired smooth muscle contractility, dysfunction of the enteric and autonomic nervous systems, and gut microbiota dysbiosis, which may promote chronic low-grade inflammation. Major contributing factors include physical inactivity, sarcopenia, dehydration, inappropriate defecation posture, and polypharmacy, particularly opioids and anticholinergic agents. Importantly, these factors interact through the brain–gut–microbiota axis, contributing not only to gastrointestinal dysfunction but also to systemic outcomes such as frailty, cognitive decline, and increased healthcare burden, thereby supporting a multidimensional disease framework. **Conclusions:** The available evidence collectively supports the plausibility of framing constipation in older adults as a systemic geriatric syndrome, though formal validation of this classification requires further longitudinal and mechanistic research. Comprehensive and individualized management strategies, extending beyond simple laxative use, are essential to reduce complications and preserve functional health in aging populations. Further studies are required to validate this framework.

## 1. Introduction

Constipation is one of the most prevalent gastrointestinal symptoms among older adults, affecting approximately 10–30% of community-dwelling individuals [1] and up to 40–60% of those residing in long-term care facilities [2]. Age-related declines in gastrointestinal motility, alterations in neural regulatory mechanisms, the presence of multiple comorbidities, and polypharmacy interact to produce a characteristic pathophysiological profile in this population [3].

With the rapid aging of the global population, constipation has become a significant contributor to healthcare utilization, including outpatient visits, laxative prescriptions, emergency department presentations, and hospital admissions [4]. In addition, constipation-related complications such as fecal impaction, bowel obstruction, and delirium are associated with increased healthcare costs and caregiver burden, making constipation an important issue from a socioeconomic perspective.

Constipation in older adults extends beyond a simple reduction in bowel movement frequency. Accumulating evidence indicates that it is closely associated with impaired quality of life (QOL), a decline in activities of daily living (ADL), and progression of frailty [5]. Moreover, constipation has been identified as a risk factor for delirium and cognitive decline [6], underscoring its clinical significance within the context of comprehensive geriatric assessment.

Traditionally, constipation has been conceptualized as a localized gastrointestinal disorder; however, this view may be insufficient to capture its full clinical and biological complexity in older adults. Increasing evidence suggests that constipation arises from multifactorial interactions involving not only gastrointestinal motility but also systemic processes such as neurocognitive regulation, immune function, and metabolic homeostasis. These interconnected mechanisms are closely linked through the brain–gut–microbiota axis, which plays a central role in integrating peripheral and central pathways. Furthermore, many of the factors associated with constipation in older adults—such as frailty, multimorbidity, polypharmacy, and functional decline—overlap substantially with those observed in established geriatric syndromes. In this context, constipation may be more appropriately understood not merely as a symptom or an isolated disorder, but as a clinical condition reflecting systemic vulnerability and impaired physiological reserve. Unlike traditional geriatric syndromes, constipation may serve not only as a consequence but also as an early clinical marker—and potentially a modifiable indicator—of systemic decline.

This narrative review aims to synthesize recent literature indexed in PubMed between 2023 and 2025, focusing on studies in which constipation and older adults were designated as major topics. Geriatric syndromes are typically defined as multifactorial conditions associated with impaired physiological reserve, functional decline, and increased vulnerability to adverse outcomes—definitions most rigorously applied to frailty, delirium, and falls. Prior narrative reviews of constipation in older adults have described its multifactorial nature, but none have systematically evaluated whether constipation meets the defining criteria of a geriatric syndrome. This review addresses that gap by proposing and applying an explicit three-criterion operational definition to the available evidence. Constipation is operationally defined as meeting the threshold for a systemic geriatric syndrome when evidence supports all three of the following: (i) multifactorial pathogenesis spanning multiple organ systems, (ii) bidirectional association with functional decline and frailty, and (iii) contribution to adverse systemic outcomes beyond the gastrointestinal tract. The review integrates current evidence on pathophysiology, contributing factors, and clinical consequences within this framework and highlights implications for comprehensive, individualized management. Unlike prior reviews that describe constipation as multifactorial without formal criteria, this framework explicitly evaluates each criterion and acknowledges where the evidence remains incomplete.

A key distinction from traditional geriatric syndromes is that constipation may function not only as a consequence of systemic vulnerability but also as an early clinical marker—and potentially a modifiable indicator—of broader physiological decline.

## 2. Methods

### 2.1. Study Design

This study was conducted as a narrative review to provide an integrated overview of recent advances in the understanding of constipation in older adults. The review focused on diagnostic criteria, epidemiological trends, pathophysiological mechanisms, and clinical implications. Rather than performing a formal systematic review or meta-analysis, we adopted a descriptive and interpretive approach to prioritize clinical relevance and practical applicability. Although a formal risk-of-bias assessment was not performed—consistent with the scope of a narrative review—the strength of evidence was qualitatively stratified by study design: randomized controlled trials and large prospective cohort studies were considered high-quality evidence; cross-sectional and retrospective studies, moderate-quality; and case series or expert opinions, low-quality. Where inconsistencies existed across studies, the direction and degree of disagreement are noted in the relevant sections. This approach allows readers to judge the weight of evidence supporting individual claims while acknowledging that a formal quantitative appraisal was beyond the scope of this review.

### 2.2. Search Strategy

A literature search was performed using the PubMed database. The search period was defined from 1 January 2023 to 31 December 2025. The following Medical Subject Headings (MeSH) terms were used:

- “Constipation” [Major Topic],
- “Aged” [MeSH Terms].

Search strategy:

(Constipation[Majr] AND Aged[MeSH Terms]) AND (2023:2025[pdat])

Only English-language articles were included.

To enhance comprehensiveness, additional relevant studies published prior to 2023 were also considered. These included seminal and “landmark” studies, defined as studies that met at least two of the following three criteria: (i) established a widely accepted pathophysiological mechanism subsequently referenced in clinical guidelines or major reviews; (ii) had been cited ≥100 times in PubMed-indexed literature; or (iii) provided a foundational epidemiological or clinical framework still referenced in recent (post-2020) reviews. These studies were identified through backward citation tracking of the primary included articles and through review of two authoritative clinical guidelines. Inclusion of pre-2023 studies was limited to cases where the evidence was necessary to contextualize current findings and was not duplicated by more recent data. The rationale for each landmark study’s inclusion is noted in the text. Such studies were identified through manual screening of reference lists of selected articles and relevant reviews, as well as through expert knowledge. The strength of evidence was qualitatively assessed based on study design (e.g., RCTs, cohort studies, cross-sectional studies). To enhance reproducibility, studies were included as “landmark” only if they met at least two of the predefined criteria.

### 2.3. Eligibility Criteria

Inclusion Criteria

Studies meeting all of the following criteria were included:

- Studies involving older adults (generally ≥65 years);
- Constipation addressed as a primary research topic;
- Original research, systematic reviews, epidemiological studies, or clinical studies;
- Availability of an abstract.

In addition, influential earlier studies were included irrespective of publication date when they met the predefined criteria for “landmark” studies and provided essential context for interpreting recent evidence.

Exclusion Criteria

Studies were excluded if they met any of the following criteria:

- Studies focusing on pediatric or younger populations;
- Studies in which constipation was evaluated only as a secondary outcome;
- Case reports, editorials, or studies available only as conference abstracts;
- Animal studies or basic experimental research without clinical relevance.

**Figure 1:**
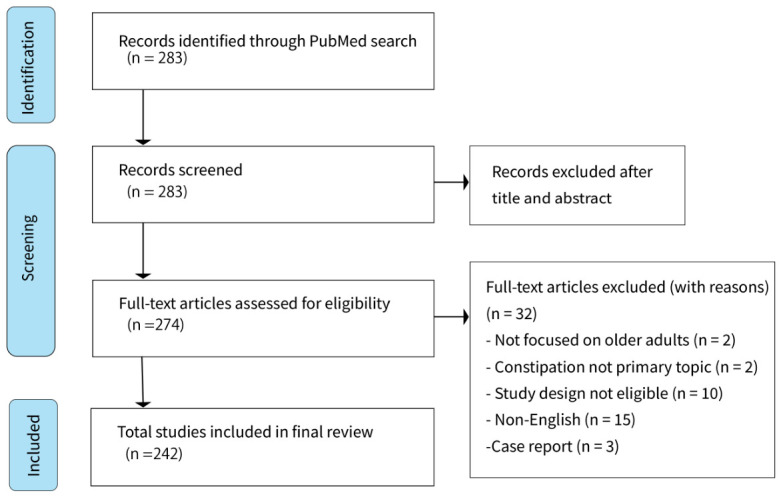
Flow diagram of the study selection process.

The numbers of studies for each exclusion reason is shown in Figure 1.

### 2.4. Figure Design and Source Attribution

All figures were created by the authors using Adobe Illustrator (v 29.8.6) for the purpose of conceptual illustration. No copyrighted materials or previously published content were used.

## 3. Definition of Constipation and Diagnostic Challenges in Older Adults

According to the Rome IV criteria, the diagnosis of functional constipation requires the presence of symptoms during the preceding three months, with symptom onset at least six months before diagnosis. At least two of the following symptoms must be present in ≥25% of defecations: fewer than three spontaneous bowel movements per week, straining during defecation, passage of hard or lumpy stools, a sensation of incomplete evacuation, or a sensation of anorectal obstruction [7].

In older adults; however, secondary causes—including medication-induced constipation, neurological disorders, and metabolic abnormalities—are highly prevalent and frequently coexist, making strict differentiation between functional and chronic constipation clinically challenging. As a result, differentiation between functional constipation and chronic constipation is often conducted, and managed under the broader category of chronic constipation using a comprehensive approach [3].

Furthermore, long-standing constipation in older adults often leads to symptom adaptation, whereby patients may underrecognize or fail to report defecatory difficulties as explicit complaints [8]. Therefore, assessment based solely on bowel movement frequency may underestimate the true burden of constipation.

Among older adults with cognitive impairment or mild cognitive decline, accurate self-reporting of symptoms is particularly difficult. In these individuals, constipation may present atypically, manifesting as non-specific symptoms such as abdominal discomfort, reduced appetite, or behavioral changes rather than direct complaints of constipation [1].

## 4. Epidemiology of Constipation in Older Adults

### 4.1. Prevalence and Age-Specific Patterns

Numerous studies have demonstrated that the prevalence of constipation increases with advancing age [9]. While the global prevalence of constipation in the general adult population is estimated to be approximately 14% [10], rates among community-dwelling adults aged 60 years and older have been reported to reach as high as 33.5% [11]. A particularly marked increase in prevalence has been observed in individuals aged 75–79 years [12], suggesting the presence of a critical age window during which age-related physiological and functional changes may substantially influence bowel function. However, reported prevalence rates vary substantially across studies, likely reflecting differences in study populations (e.g., community-dwelling vs. institutionalized older adults), diagnostic criteria (Rome IV vs. self-report vs. laxative use as proxy), and geographic and cultural factors. These methodological variations should be considered when interpreting epidemiological estimates. These variations limit direct comparability across studies and highlight the need for standardized diagnostic criteria.

### 4.2. Differences Between Community-Dwelling and Institutionalized Older Adults

Living environment plays a significant role in the development of constipation among older adults. Compared with community-dwelling older individuals, residents of long-term care facilities consistently exhibit a higher prevalence of constipation [2]. In institutionalized settings, age-related physiological changes are compounded by reduced physical activity, polypharmacy, insufficient dietary fiber and fluid intake, as well as cognitive impairment and psychological factors, all of which interact to exacerbate constipation [8].

### 4.3. Female Predominance and Age-Related Changes

One of the most prominent epidemiological features of constipation is its sex-related difference. Across most age groups, the prevalence of constipation in women is approximately twice that in men [13]. This disparity has been attributed to the effects of sex hormones, such as progesterone and estrogen, which are known to slow gastrointestinal transit, as well as anatomical and functional differences in the pelvic floor [13].

Although female predominance is evident in younger and middle-aged populations, this sex difference tends to diminish with advancing age, suggesting that hormonal influences play a key role in the development and age-related variation in constipation [14].

### 4.4. Geographic and Socioeconomic Factors

Geographic variation in constipation prevalence has been reported; however, findings remain inconsistent, likely reflecting differences in cultural background, dietary habits, and study methodologies [15]. Socioeconomic factors, such as lower socioeconomic status, lower educational attainment, and dietary patterns such as low fiber intake, have been identified as important risk factors for constipation [13].

Prevalence also varies across regions, ranging from approximately 16% in the United States to 8–26% in Europe [16]. These differences may be explained by regional variations in diet, physical activity levels, and cultural perceptions of bowel habits. Notably, inconsistencies across regions may also arise from heterogeneity in study design, dietary assessment methods, and definitions of constipation, limiting direct comparability between studies.

## 5. Pathophysiology

### 5.1. Peripheral Mechanisms (Smooth Muscle, ICC, Anorectal Function)

One of the key pathophysiological mechanisms underlying constipation in older adults is age-related structural degeneration of the gastrointestinal tract, accompanied by impaired neuromuscular control of motility. Aging is associated with a reduction in both the density and contractile capacity of colonic smooth muscle cells. In addition, decreased vascular elasticity reduces intestinal blood supply, which contributes to impaired colonic peristalsis and absorptive function [17].

Beyond smooth muscle alterations, aging is also associated with a decline in the number of interstitial cells of Cajal (ICCs), which function as pacemaker cells regulating gastrointestinal motility [18]. A reduction in ICCs disrupts the coordination of peristaltic activity and weakens propulsive contractions, thereby prolonging colonic transit time in older adults [18].

Anorectal functional changes further contribute to the development of constipation. Compared with younger individuals, older adults exhibit significantly reduced anal resting pressure (MARP/MERP) and maximum squeeze pressure (MSP) [14]. These changes are attributed to age-related atrophy of the external anal sphincter, loss of muscle fibers, and increased connective tissue deposition, all of which reduce sphincter tone and contractile strength [19]. Furthermore, age-related neuronal loss diminishes rectal sensitivity, resulting in an elevated sensory threshold for the urge to defecate and a blunting of the defecation reflex [20].

In addition, age-related weakening and impaired coordination of the pelvic floor musculature become increasingly evident. These alterations contribute to rectoanal dyssynergia during defecation and represent a major component of defecatory disorders and outlet-type constipation in older adults [21].

### 5.2. Central and Autonomic Dysregulation (CNS–ENS–ANS)

Constipation in older adults reflects dysfunction of an integrated regulatory network linking the central nervous system (CNS), enteric nervous system (ENS), and autonomic nervous system (ANS), collectively referred to as the central–enteric–autonomic axis. Rather than being solely a peripheral disorder, age-related constipation arises from multilevel neural dysregulation affecting both motor and sensory components of defecation [22].

Within the intestinal wall, the Auerbach’s (myenteric) plexus functions as a critical regulator of peristalsis. However, aging is associated with structural and functional degeneration of the ENS [23]. Specifically, older adults exhibit a reduction in enteric neurons and decreased expression of connexin 43, a key gap-junction protein essential for intercellular signal transmission [24]. These degenerative changes disrupt the coordination of intestinal motility and contribute to prolonged colonic transit time (CTT) [23].

The autonomic nervous system serves as a key pathway for transmitting central signals to the gastrointestinal tract and maintaining homeostasis. Sympathetic overactivity, often associated with stress or anxiety, suppresses gastrointestinal motility, thereby exacerbating constipation [25]. In contrast, the vagus nerve, the principal parasympathetic pathway, enhances gut motility, sensory processing, and anti-inflammatory responses. In older adults, autonomic dysregulation—characterized by reduced heart rate variability and diminished vagal tone—is frequently observed, contributing to impaired gastrointestinal regulation [26].

Dysfunction of the neural axis affects not only motor function but also sensory processing. Rectal hyposensitivity, defined as reduced perception of rectal distension, is commonly observed in older adults with chronic constipation [27]. When rectal sensory signals fail to reach the brain accurately, the urge to defecate diminishes, leading to fecal retention and progressive rectal dilation. This process further impairs rectal sensitivity and establishes a self-perpetuating vicious cycle [27].

Thus, constipation in older adults is fundamentally characterized by multilayered dysfunction of the neural axis, spanning from peripheral enteric degeneration to impaired central regulation of defecation. This framework highlights the need for integrative therapeutic approaches targeting the brain–gut–autonomic network.

### 5.3. Gut Microbiota and Inflammaging

In recent years, increasing attention has been directed toward the role of gut microbiota in the pathophysiology of constipation in older adults. Studies using 16S rRNA sequencing have demonstrated that older adults with functional constipation exhibit altered microbial composition and reduced diversity compared with healthy individuals [28]. Specifically, shifts in microbial populations—including reductions in beneficial taxa and relative increases in genera such as Bacteroides, Prevotella, and Ruminococcus—have been reported, reflecting constipation-associated dysbiosis [28].

Aging itself is associated with a decline in microbial diversity and a reduction in short-chain fatty acid (SCFA)-producing bacteria, such as *Faecalibacterium prausnitzii*, which play a crucial role in maintaining intestinal and metabolic homeostasis [29]. These microbiota changes influence host immune function and mucosal immune responses, contributing to intestinal barrier dysfunction and increased permeability.

Dysbiosis may promote chronic low-grade systemic inflammation (“inflammaging”), which is increasingly recognized as a key mechanism underlying geriatric syndromes, including constipation. These processes link gut microbiota alterations to systemic physiological decline and functional impairment in older adults [30].

Although multiple studies have reported associations between gut microbiota alterations and constipation in older adults, the specific microbial signatures are not entirely consistent. These discrepancies likely reflect differences in geographic population characteristics, dietary habits, and comorbidity profiles. Importantly, most evidence derives from cross-sectional studies with small samples, limiting causal inference. The few available longitudinal intervention studies suggest that microbiota modulation can improve constipation symptoms, but effect sizes are modest, and durability beyond the intervention period is unclear. Larger, well-characterized longitudinal cohorts are needed to establish whether dysbiosis precedes constipation or results from it.

## 6. Contributing Factors to Constipation in Older Adults

### 6.1. Physical and Nutritional Factors

Restricted physical activity and a sedentary lifestyle are major contributors to constipation in older adults [8]. Prolonged bed rest, particularly lasting longer than 48 h, has been identified as an independent risk factor, significantly increasing the likelihood of constipation [31]. Reduced physical activity decreases intestinal smooth muscle contractility, resulting in delayed colonic transit [31]. In addition, loss of skeletal muscle mass (sarcopenia) reduces intra-abdominal pressure during defecation, thereby impairing effective bowel evacuation [32].

Older adults are particularly susceptible to dehydration due to a diminished thirst response, frequent use of diuretics, and intentional fluid restriction. Reduced intraluminal water content leads to hard stools and increased difficulty with defecation [33]. Inadequate intake of dietary fiber and fluids is common and represents a key modifiable risk factor [34]. Furthermore, chronic malnutrition, including hypoalbuminemia, contributes to mucosal atrophy and neuromuscular dysfunction, resulting in impaired gastrointestinal motility [20]. While dietary fiber and fluid intake are widely recommended, clinical responses may vary among individuals, particularly in frail or malnourished older adults, suggesting that uniform recommendations may not be universally effective.

### 6.2. Medication-Related Factors

#### 6.2.1. Opioid Analgesics

Older adults frequently have multiple chronic conditions, resulting in widespread polypharmacy, which represents a major modifiable contributor to constipation [35]. Drug-induced constipation occurs through suppression of gastrointestinal motility and alteration in stool consistency, making it one of the most important reversible causes of constipation [35].

Opioids are indispensable for pain management but are strongly associated with opioid-induced constipation (OIC) [36]. These agents bind to μ-opioid receptors in the enteric nervous system, inhibiting peristalsis and increasing intestinal water absorption, which leads to harder stools and impaired stool passage [37].

The risk of constipation varies depending on opioid type and dosage. Compared with codeine, morphine, oxycodone, and fentanyl are associated with a higher risk of severe constipation, whereas tramadol is associated with a relatively lower risk, likely due to its additional serotonergic and noradrenergic mechanisms. Furthermore, higher opioid doses further increase constipation risk [37].

#### 6.2.2. Anticholinergic Medications

Anticholinergic agents inhibit parasympathetic activity and reduce gastrointestinal motility, thereby promoting constipation. Drugs used for urinary symptoms may simultaneously impair intestinal motility, exacerbating constipation [38]. In addition, many psychotropic medications possess anticholinergic properties, increasing the risk of chronic constipation with long-term use [36]. In patients with Parkinson’s disease, antiparkinsonian medications may further aggravate constipation by interacting with underlying enteric neurodegeneration and altering gut motility and microbiota composition [39].

#### 6.2.3. Other Medications and Gastrointestinal Effects

Long-term use of proton pump inhibitors (PPIs) may indirectly contribute to constipation through alterations in the gut environment [40]. PPI use has been associated with increased intestinal permeability markers, such as α1-antitrypsin (AAT), suggesting impairment of intestinal barrier function [40]. In addition, elevated gastric pH may promote fungal overgrowth and microbial imbalance, thereby contributing to dysbiosis [40]. This altered microbial environment may, in turn, promote chronic low-grade inflammation (“inflammaging”) and disrupt gastrointestinal motility [40]. Calcium channel blockers, which are commonly prescribed for hypertension, inhibit smooth muscle contraction and are recognized risk factors for constipation [16].

Importantly, even medications used to treat constipation may contribute to disease perpetuation when used chronically. Long-term use of stimulant laxatives has been associated with tolerance, dependence, and reduced intrinsic colonic motility, thereby creating a vicious cycle of worsening constipation [41,42]. Chronic use may also alter gut microbiota composition, reduce microbial diversity, and impair mechanisms regulating stool hydration and motility, further exacerbating constipation [8]. In addition, prolonged use of anthraquinone-containing laxatives (e.g., senna, aloe, rhubarb) may induce colonic melanosis and impair enteric nervous system function, resulting in reduced colonic contractility [34].

### 6.3. Environmental and Social Factors

Environmental and social factors play a critical role in modulating constipation risk in older adults, particularly through restricted access to toileting, loss of independence, and suboptimal defecation posture [43]. These factors interact with age-related physical decline to exacerbate functional constipation [43].

In patients with chronic obstructive pulmonary disease (COPD), reduced physical activity compared with age-matched healthy individuals has been associated with a higher prevalence of constipation, with reported rates of approximately 40% [44].

Physiologically optimal defecation postures include the seated or squatting position, which facilitates defecation by relaxing the puborectalis and pelvic floor muscles and straightening the anorectal angle [27]. In contrast, non-physiological postures, such as lateral decubitus positioning during bedpan use, impair anorectal pressure and increase the risk of defecatory dysfunction [27].

Increasing care dependency and loss of independence are closely associated with constipation in older adults [45]. Individuals requiring partial or full assistance with feeding or toileting exhibit a higher prevalence of functional constipation compared with independent individuals [45]. Furthermore, limited access to toilets and habitual suppression of defecation weaken the defecation reflex and promote chronic constipation [46].

Increased dependency often restricts both mobility and timely access to toileting, thereby amplifying the impact of underlying physiological decline [46]. A prospective study of community-dwelling older adults aged ≥75 years demonstrated that sarcopenia is an independent risk factor for incident constipation, highlighting the importance of maintaining muscle strength and functional independence [24].

## 7. Clinical Impact

### 7.1. Cognitive, Psychological, and Systemic Outcomes

Recent evidence indicates that constipation in older adults is closely associated not only with physical health but also with cognitive function and psychological well-being [1]. Large-scale epidemiological studies have demonstrated a significant association between constipation and cognitive impairment, including mild cognitive impairment (MCI) and dementia [1]. In particular, the “body-first” hypothesis proposes that constipation may represent an early prodromal manifestation of neurodegenerative disorders, especially in non-amnestic MCI [1].

Chronic constipation, particularly when characterized by reduced bowel movement frequency, has also been independently associated with an increased risk of incident MCI in older adults [1]. These associations are thought to be mediated, at least in part, by disruption of the brain–gut–microbiota axis, linking chronic constipation to neuroinflammation and increased vulnerability to cognitive decline [47].

Constipation-associated dysbiosis may impair intestinal barrier integrity and increase intestinal permeability (“leaky gut”), allowing bacterial metabolites and endotoxins to enter the systemic circulation [48]. These circulating factors may subsequently affect the central nervous system through increased blood–brain barrier permeability, thereby promoting neuroinflammatory processes [48,49].

Furthermore, individuals with constipation exhibit reduced production of short-chain fatty acids (SCFAs), which play a critical role in maintaining intestinal and blood–brain barrier integrity, regulating immune responses, and exerting neuroprotective effects [50]. Reduced SCFA production may therefore enhance neuroinflammation and oxidative stress, ultimately contributing to cognitive deterioration in older adults [50].

A bidirectional relationship between constipation and depressive symptoms has also been reported [51]. Patients with defecatory disorders exhibit a high prevalence of psychological comorbidities, with depressive symptoms observed in approximately 82.5% and anxiety symptoms in 55.6%. Greater severity of defecatory symptoms has been shown to correlate with increased psychological distress [52]. In hospitalized or postoperative older adults, constipation has gained clinical attention as a secondary precipitating factor for delirium, alongside urinary retention and pain [31]. Severe complications, including fecal impaction, bowel obstruction (ileus), rectal ulcers, and stercoral perforation, may occur if constipation is left untreated [42]. In addition, in patients with acute heart failure or following myocardial infarction, the presence of constipation has been associated with increased risks of rehospitalization and mortality [53].

### 7.2. Prognosis and Healthcare Burden

If inadequately managed, chronic constipation in older adults may lead to serious complications and significantly increase healthcare burden [51]. Untreated constipation can result in fecal impaction, stercoral ulcers, diverticulitis, and even intestinal perforation, all of which represent major causes of hospitalization in older populations [51]. Among hospitalized older patients, constipation has been associated with increased risks of delirium, intensive care unit (ICU) admission, and cardiopulmonary arrest [54]. In addition, patients with constipation exhibit higher rates of hospital readmission within 90 days, highlighting the importance of effective long-term management strategies [54].

The economic burden of constipation is substantial. In the United States, the annual healthcare cost per patient with chronic constipation has been estimated at approximately USD 11,991, including outpatient visits, hospitalizations, and costs associated with treatment failure [43]. Another report estimated that constipation-related medical consultations account for approximately 2.5 million visits annually, with an average cost of USD 2752 per patient [55].

**Figure 2:**
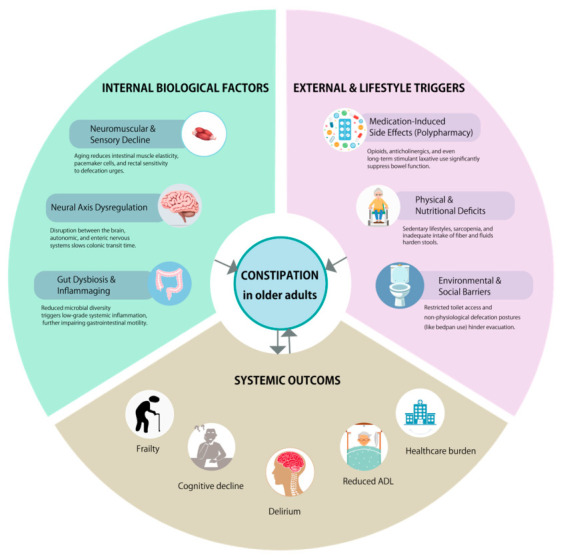
Constipation as a systemic geriatric syndrome: an integrated pathophysiological framework.

Constipation also impairs daily functioning and contributes to functional decline and loss of independence, thereby increasing reliance on caregiving. Fecal incontinence associated with constipation is one of the leading reasons for institutionalization among older adults [56]. In severe cases, conventional treatments such as laxatives or enemas may be ineffective, requiring manual disimpaction, which imposes a significant physical and psychological burden on both patients and caregivers [57]. Figure 2 illustrates this proposed integrative framework, highlighting the multidimensional interactions underlying constipation in older adults.

## 8. Management Strategies

### 8.1. Non-Pharmacological Interventions

#### 8.1.1. Dietary Modification

In the management of constipation in older adults, dietary intervention is recommended as the first-line strategy because it carries a low risk of adverse effects and promotes patient autonomy [42]. Diet plays a central role not only in stool formation but also in regulating the composition and function of the gut microbiome, which is increasingly recognized as a key determinant of gastrointestinal and systemic health.

Dietary fiber increases stool bulk and improves the frequency of bowel movements. A daily intake of 25–35 g is generally recommended [58], and prunes (dried plums) have been shown to be particularly effective—containing both fiber and sorbitol—significantly improving QOL and subjective satisfaction [8]. Furthermore, a dietary pattern (DI-GM) characterized by increased consumption of whole grains, fermented dairy products, vegetables, coffee, and green tea, while reducing red meat and high-fat foods, promotes microbial diversity and lowers the risk of constipation [59].

Beyond fiber, optimal protein intake is essential for maintaining gastrointestinal homeostasis in older adults. Recent cross-sectional data indicate that high-protein diets, along with the excessive consumption of sweet pastries and dairy products, are associated with an increased risk of digestive disorders [60]. Conversely, the intake of legumes and fiber is reported to reduce the frequency of gastrointestinal symptoms [60].

A key mechanistic link between diet, the microbiome, and host physiology is the metabolism of L-tryptophan (TRP), an essential amino acid obtained from the diet [61]. More than 90% of TRP is metabolized within the gastrointestinal tract, where it is distributed across three major metabolic pathways that compete for substrate availability [62]. The balance among these pathways is strongly influenced by both dietary composition and the gut microbiome and plays a critical role in functional constipation (FC) and gut–brain axis regulation [61].

1. Serotonin (5-HT) pathway: Approximately 1–2% of dietary TRP is metabolized via this pathway, initiated by tryptophan hydroxylase 1 (TPH-1) in enterochromaffin cells [62]. Serotonin directly stimulates intestinal motility and regulates secretion. Clinically, older adults with FC and postmenopausal women often exhibit reduced urinary levels of 5-hydroxyindoleacetic acid (5-HIAA), a serotonin metabolite, which correlates with symptom severity [61]. Enhancement of this pathway, for example through TRP supplementation, may improve bowel function [62].
2. Kynurenine (KYN) pathway: This is the dominant metabolic route, accounting for approximately 95% of TRP metabolism and initiated by indoleamine 2,3-dioxygenase (IDO-1), which is upregulated by inflammatory signals and microbial stimuli [62]. While some downstream metabolites are neuroprotective, others, such as quinolinic acid, exert neurotoxic effects at high concentrations [62]. In constipation, overactivation of this pathway may reduce TRP availability for serotonin synthesis, thereby impairing intestinal motility and potentially contributing to mood disturbances [61].
3. Indole pathway: Approximately 2–3% of TRP is metabolized by gut bacteria via tryptophanase into indole, which is subsequently converted in the liver to indoxyl sulfate [62]. Indican is a clinically relevant biomarker of intestinal dysbiosis and is often elevated in patients with constipation, correlating with symptom severity [61]. At high concentrations, it may exert cytotoxic and neurotoxic effects, disrupt the gut–brain axis, and contribute to psychological symptoms or cognitive decline [61].

The balance among these TRP metabolic pathways is therefore a critical determinant of both gastrointestinal function and neuropsychological health in older adults. Clinical studies have shown that supplementation with L-tryptophan (e.g., 1000 mg/day) in combination with a low fermentable oligosaccharides, disaccharides, monosaccharides and polyols (FODMAP) diet can enhance serotonin production and improve constipation symptoms [62]. In addition, functional foods such as dark tea, including preparations fermented with Lactobacillus paracasei, may improve constipation by modulating gut microbiota composition, enhancing intestinal barrier function, and influencing TRP metabolism [63].

Finally, comprehensive dietary management should consider oral and functional status. A decline in chewing ability, assessed by functional tooth units, leads to reduced intake of fiber-rich foods and impaired digestive efficiency, thereby increasing the risk of constipation [64]. Therefore, dental rehabilitation and maintenance of oral function are important components of integrated care in older adults [64].

Despite supportive evidence, the overall quality of evidence for dietary interventions in older adults remains moderate to low. Most studies are short-term (≤12 weeks), small-scale (*n* < 100), and rely on self-reported outcomes. RCT evidence is strongest for polyethylene glycol and certain probiotics; evidence for dietary fiber, prune consumption, and the DI-GM pattern derives largely from observational or non-randomized studies and may not generalize to frail or malnourished older adults. No head-to-head comparisons between dietary strategies in this population have been published. Future large-scale RCTs with standardized outcomes and longer follow-up are needed.

#### 8.1.2. Physical Activity and Adjunctive Mechanical Interventions

Physical activity, particularly walking, stimulates gastrointestinal motility and improves bowel function. In older adults, walking more than 5500 steps per day has been associated with improved stool consistency and bowel habits [65]. Non-invasive interventions, such as abdominal vibration combined with walking exercise, have been shown to enhance intestinal motility and facilitate defecation safely and effectively [65]. In addition, abdominal massage and thermal therapy have demonstrated beneficial effects on bowel frequency and stool consistency [66,67]. Emerging non-pharmacological devices, including vibration capsules, are gaining attention as novel therapeutic options that may reduce reliance on pharmacological treatments [68].

#### 8.1.3. Toileting Behavior, Posture, and Environmental Modifications

Environmental factors play a critical role in facilitating effective defecation, particularly in older adults with cognitive impairment [2]. Establishing a regular morning bowel routine that utilizes the gastrocolic reflex has been identified as a protective factor against functional constipation [45]. Adopting a semi-squatting posture (e.g., using a footstool to elevate the knees above hip level) helps straighten the anorectal angle and reduce excessive straining, thereby facilitating defecation [69]. Sleep disturbances and irregular sleep patterns may negatively affect gut microbiota composition and autonomic nervous system function, contributing to worsening constipation [70]. Therefore, individualized toileting support aligned with the patient’s daily rhythm, along with a comfortable and private environment, is essential to reduce psychological barriers to defecation [16].

### 8.2. Pharmacological Therapy

#### 8.2.1. Conventional Laxatives: Efficacy and Limitations

Despite the availability of new pharmacological agents, more than 60% of patients continue to experience persistent symptoms, indicating the limitations of conventional therapies [44]. Osmotic laxatives, including magnesium oxide, sorbitol, and polyethylene glycol (PEG), act by retaining water in the intestinal lumen, thereby softening stools [71,72]. However, magnesium oxide requires caution in older adults and those with renal dysfunction due to the risk of hypermagnesemia, which may adversely affect cardiac function [33].

Stimulant laxatives, such as bisacodyl, senna, and rhubarb, directly stimulate intestinal peristalsis [36,67]. While effective in the short term, long-term use may result in tolerance, dependence, and colonic melanosis, potentially worsening constipation. Therefore, their use should be limited to short-term or intermittent administration [42].

#### 8.2.2. Novel Secretagogues and Prokinetic Therapies

Elobixibat inhibits bile acid reabsorption in the ileum, increasing bile acid delivery to the colon and thereby promoting water secretion and colonic motility [73]. In Japanese healthcare settings, it has demonstrated favorable cost-effectiveness compared with other treatments [74]. Lubiprostone activates ClC-2 chloride channels, enhancing intestinal fluid secretion, although adverse effects such as nausea and rare ischemic colitis have been reported, requiring careful monitoring [74]. Guanylate cyclase-C (GC-C) agonists, such as linaclotide, improve bowel function by increasing intestinal secretion and reducing visceral hypersensitivity [73].

Although pharmacological therapies provide symptomatic relief, comparative effectiveness across agents is insufficiently established, particularly in older adults with multimorbidity, who are consistently underrepresented in clinical trials. PEG has the most robust RCT evidence for chronic constipation in older adults and is generally preferred over stimulant laxatives for long-term use. Prucalopride has demonstrated efficacy in RCTs, but most trials excluded patients with severe renal impairment or cardiac comorbidities. Elobixibat data derive predominantly from Japanese populations, limiting generalizability.

#### 8.2.3. Management of Secondary and Drug-Induced Constipation

Opioid-induced constipation represents a major clinical challenge in older adults receiving chronic pain management. Peripherally acting μ-opioid receptor antagonists (PAMORAs), such as naldemedine and methylnaltrexone, have been shown to effectively improve bowel function without compromising analgesic efficacy [36].

Prucalopride, a selective 5-hydroxytryptamine (5-HT4) receptor agonist, has demonstrated higher treatment persistence and adherence compared with other agents, likely due to its once-daily dosing and favorable safety profile [75].

### 8.3. Microbiota-Targeted Therapy

Microbiota-targeted therapies, including probiotics and synbiotics, have emerged as promising options for the management of constipation in older adults due to their favorable safety profiles and potential disease-modifying effects [76]. These interventions act by modulating gut microbiota composition, enhancing short-chain fatty acid (SCFA) production, and improving intestinal barrier function, thereby influencing both gastrointestinal motility and systemic inflammation [77].

Patients with constipation often exhibit dysbiosis characterized by reduced microbial diversity, decreased SCFA-producing bacteria, and increased methanogenic species, all of which contribute to delayed intestinal transit [78]. Restoration of microbial balance through probiotic supplementation has been shown to increase beneficial genera such as *Bifidobacterium* and *Lactobacillus*, leading to improvements in stool frequency and consistency [78].

Clinical evidence supports the efficacy of these approaches in older populations. Probiotic supplementation with *Lactiplantibacillus plantarum* strains has been shown to improve bowel movement frequency, stool consistency, and perceived stress in adults aged 50–85 years [76]. In institutionalized older adults, daily intake of *Bifidobacterium longum* and *Bifidobacterium lactis* significantly increased the proportion of normal bowel movements [2]. Similarly, supplementation with *Weizmannia coagulans* BC01 improved stool consistency and QOL, accompanied by increased SCFA production [78]. In postoperative older patients, *Lactobacillus rhamnosus* JYLR-127 has been reported to improve bowel frequency and reduce abdominal symptoms [77].

Synbiotics, combining probiotics with prebiotics such as fructooligosaccharides (FOS), may exert synergistic effects on gut microbiota and bowel function. A randomized controlled trial in adults aged ≥60 years demonstrated that *Bifidobacterium animalis* subsp. *lactis* BL-99 combined with FOS significantly increased spontaneous bowel movements and reduced whole-gut transit time, with sustained effects after treatment cessation [79].

In addition to gastrointestinal effects, microbiota-targeted therapies may influence the gut–brain axis, particularly in patients with neurological conditions. In Parkinson’s disease, multi-strain probiotic supplementation improved constipation symptoms while reducing systemic inflammation and enhancing treatment response [39].

However, clinical responses vary depending on baseline microbiota composition, comorbidities, and lifestyle factors, highlighting the importance of individualized therapeutic strategies [80]. Table 1 provides a comparative overview of major therapeutic modalities for constipation in older adults, focusing on their mechanisms of action, elderly-specific benefits, risks, and clinical considerations.

Although these findings are promising, several limitations constrain interpretation. First, most trials use single strains or strain combinations that are not comparable across studies; no head-to-head trials between strains or products have been published in older adult populations. Second, trial durations are short (typically 4–8 weeks), with limited follow-up after cessation. Third, outcome measures are heterogeneous (stool frequency, consistency, transit time, or QOL scores), precluding meta-analytic synthesis. Fourth, patients with severe frailty or cognitive impairment are usually excluded, limiting generalizability to the most vulnerable older adults. Taken together, the evidence supports probiotics and synbiotics as safe adjuncts but stops short of establishing them as first-line therapy without further confirmatory trials.

**Figure 3:**
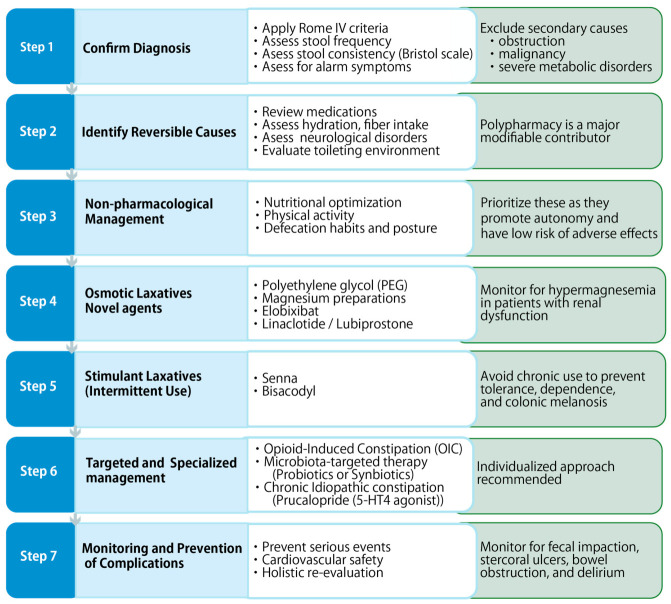
Conceptual framework for the management of constipation in older adults.

Rather than proposing a rigid treatment algorithm, we present a conceptual framework that organizes current evidence into a stepwise clinical approach for constipation management in older adults (Figure 3).

## 9. Conclusions

The evidence reviewed in this article supports the plausibility of conceptualizing constipation in older adults as a systemic geriatric syndrome, though this classification should be regarded as a hypothesis-generating framework rather than a definitive conclusion. Against the three operational criteria proposed in this review, the available evidence is strongest for multifactorial pathogenesis (criterion i), where gastrointestinal, neurological, microbiological, and behavioral components are well documented. Evidence for criterion ii (association with functional decline and frailty) is supported primarily by large cross-sectional and prospective epidemiological studies, though the bidirectionality of this relationship requires further longitudinal confirmation. Evidence for criterion iii (contribution to adverse systemic outcomes) is supported by observational data linking constipation to delirium, cognitive impairment, and increased healthcare utilization, but causal inference is limited by confounding and reverse causation. Accordingly, this framework positions constipation as a strong candidate for classification as a systemic geriatric syndrome while acknowledging that formal validation awaits future prospective and mechanistic research. This perspective highlights the need for integrated and multidimensional management strategies rather than symptom-focused treatment alone.

Constipation in older adults exhibits characteristics consistent with a systemic geriatric syndrome, arising from the complex interplay of neuromuscular dysfunction, gut microbiota alterations, lifestyle factors, and polypharmacy. The available evidence demonstrates associations with frailty, cognitive decline, psychological distress, and increased healthcare burden, underscoring its clinical and societal significance. At the same time, most of these associations derive from observational data, and causal directionality has not been fully established. This review therefore frames the systemic geriatric syndrome concept as a clinically useful working model that organizes evidence across domains and guides comprehensive management, rather than as a definitive classification.

Recent advances have improved our understanding of the underlying mechanisms, particularly the roles of the brain–gut–microbiota axis and chronic low-grade inflammation (“inflammaging”), which provide a unifying framework linking gastrointestinal dysfunction to systemic and neurocognitive outcomes. These insights suggest that constipation may serve as a potential early indicator of broader physiological decline.

From a therapeutic perspective, management should extend beyond symptomatic relief. Comprehensive and individualized strategies, incorporating lifestyle modification, optimization of pharmacological therapy, and emerging microbiota-targeted interventions, are essential for effective care. In particular, prioritizing modifiable factors and tailoring interventions to functional and cognitive status may improve both gastrointestinal and overall health outcomes in older adults.

Future research should focus on the development of age-specific diagnostic criteria, longitudinal studies evaluating long-term outcomes such as frailty and cognitive decline, and the validation of multidisciplinary management approaches. A deeper understanding of the bidirectional interactions between the gut, brain, and systemic health will be critical for advancing both preventive and therapeutic strategies. At present, heterogeneity in study design and limited high-quality longitudinal data remain important challenges, underscoring the need for more rigorous and standardized research in this field.

In conclusion, appropriate recognition and management of constipation in older adults remain essential. However, current evidence is insufficient to definitively classify constipation as a systemic geriatric syndrome. Instead, this framework should be considered hypothesis-generating and requires further validation. Further longitudinal and mechanistic studies are required to clarify its role within the spectrum of geriatric syndromes.

## Figures and Tables

**Table 1:** Summary of Treatment Options for Constipation in Older Adults and Their Clinical Implications.

Aspect	Non-Pharmacological Interventions	Pharmacological Therapy	Microbiota-Targeted Therapy
Primary Methods	Dietary fiber and fluid intake, abdominal massage (Thai, Swedish), postural adjustment, and vibrating capsules [8].	Osmotic laxatives (Mg Oxide, PEG), stimulants (Senna), prosecretory agents (Elobixibat, Lubiprostone, Linaclotide), and PAMORAs (Naldemedine) [74].	Probiotics (L. plantarum, W. coagulans) and synbiotics (Bifidobacteria + FOS) [76].
Mechanism of action	Mechanical stimulation of colonic motility, induction of the gastrocolic reflex, and optimization of the anorectal angle [67].	Increases intestinal fluid secretion via ion channels or triggers direct peristaltic stimulation via enteric nerves [74].	Reshaping gut microbiota, producing Short-Chain Fatty Acids (SCFAs), and strengthening the intestinal barrier [76].
Elderly-Specific Benefits	Enhances patient autonomy, reduces psychological distress (anxiety/depression), and provides sensory stimulation [8].	Provides rapid symptom relief; specific agents like PAMORAs are highly effective for opioid-induced constipation (OIC) [11].	Improves stool consistency and frequency sustainably while positively influencing the brain–gut microbiota axis to reduce stress [76].
Risks and Side Effects	Requires caregiver training for massage; effectiveness of exercise/diet alone can be limited by immobility [4].	Risk of dependence, tolerance, electrolyte imbalance, and hypermagnesemia in patients with renal impairment [8].	Generally safe and well-tolerated; however, some patients may experience transient diarrhea during the early evacuation of accumulated stool [72].
Economic and Clinical Utility	Offers low-cost interventions that can be integrated into primary care to reduce pharmacological dependence [8].	Elobixibat is associated with lower total costs and better QoL in Japan; productivity loss is a major indirect cost [73].	Requires additional supplement costs but can contribute to long-term health maintenance and reduced medication burden [76].

## Data Availability

Not applicable.
